# Worrying Thoughts Limit Working Memory Capacity in Math Anxiety

**DOI:** 10.1371/journal.pone.0165644

**Published:** 2016-10-27

**Authors:** Zhan Shi, Peiru Liu

**Affiliations:** 1 School of Education, China West Normal University, No 1 Shi Da Road, Nanchong City, Sichuan Province, China; 2 Key Laboratory of Child Development and Learning Science, Southeast University, Research Center for Learning Science, Ministry of Education, Nanjing, China; Center for BrainHealth, University of Texas at Dallas, UNITED STATES

## Abstract

Sixty-one high-math-anxious persons and sixty-one low-math-anxious persons completed a modified working memory capacity task, designed to measure working memory capacity under a dysfunctional math-related context and working memory capacity under a valence-neutral context. Participants were required to perform simple tasks with emotionally benign material (i.e., lists of letters) over short intervals while simultaneously reading and making judgments about sentences describing dysfunctional math-related thoughts or sentences describing emotionally-neutral facts about the world. Working memory capacity for letters under the dysfunctional math-related context, relative to working memory capacity performance under the valence-neutral context, was poorer overall in the high-math-anxious group compared with the low-math-anxious group. The findings show a particular difficulty employing working memory in math-related contexts in high-math-anxious participants. Theories that can provide reasonable interpretations for these findings and interventions that can reduce anxiety-induced worrying intrusive thoughts or improve working memory capacity for math anxiety are discussed.

## Introduction

Mathematics anxiety (MA) is defined as a feeling of tension and anxiety that interferes with the manipulation of numbers and the solving of mathematical problems in a wide variety of ordinary life and academic situations [1]. Apart from enjoying mathematics less, having lower perceptions of their mathematical abilities, and ignoring the value of mathematics in everyday life [2–4], people with MA are characterized by increased worries about math failure [5], an avoidance of math and/or numerical tasks [6], and even a negative emotional response to the prospect of doing math [4]. Despite normal performance in most thinking and reasoning tasks, individuals with MA perform poorly when numerical information is involved [3, 4, 7, 8]. Specifically, empirical evidence has been found for performance differences as a function of MA. These differences typically are not observed when engaged in simple addition or multiplication but are observed when engaged in more difficult arithmetic problems involving carry operation [9, 10]. Executing the carry operation is thought to be controlled by working memory (WM) [11, 12], which is defined as a limited resource cognitive system used to actively maintain information in the face of ongoing processing and/or distraction [13–17]. A large number of theoretical and empirical studies have thus developed a generally accepted interpretation of the effect of MA on mathematics, which claims that MA detrimentally impacts mathematical performance by disrupting WM resources otherwise devoted to skill execution [18–22]. For example, in a seminal study investigating the relationship between MA and WM, high-math-anxious (HMA) and low-math-anxious (LMA) participants were asked to perform two-column addition problems requiring a carry operation, which placed a load on WM [18]. Participants performed these problems while completing a secondary letter memory task involving the maintenance of either 2-letter strings or 6-letter strings in memory. Math error rates under the 2-letter load for HMA participants were only slightly larger than LMA participants. However, HMA participants produced significantly more math errors under the 6-letter load than for LMA participants. Based on processing efficiency theory (PET) [20], Ashcraft and Kirk [18] concluded that the high letter load was detrimental to HMA participants’ WM performance because their worries about math disrupted WM resources that might otherwise have been available to perform the difficult math problems. However, as admitted by Ashcraft and Kirk [18], they “do not test the specifics of Eysenck and Calvo’s prediction [20] here, which states that it is intrusive thoughts and worry (in this case, about math) that detract from available working memory capacity (WMC).” Instead, they “assess the more general prediction that MA disrupts WM processing when the cognitive task involves arithmetic or math-related processes” (p. 226) [18].

Worrying intrusive [20, 23, 24] thoughts compete for processing resources [25,26]. This processing can be measured using reading span (RS) tasks, a well-developed paradigm of WMC, interleaving the presentation of to-be-remembered target stimuli, such as words or letters, with the presentation of a demanding, secondary processing task, such as comprehending sentences [14]. Conway et al. [14], in their methodological review of these tasks, stated that, “Attention is often captured by events in the environment and by thoughts that intrude into consciousness. Those perceptions and thoughts, in turn, lead inexorably to other thoughts. However, the solution to life’s problems often requires that such automatically elicited thoughts, associations, and captured attention be resisted and thought be directed or controlled. We have argued that this ability to control attention and thought represents the common construct measured by tests of WMC” (p. 777). However, as traditional RS tasks require short-term retention of emotionally-neutral information while simultaneously processing competing emotionally-neutral information, it is difficult for any such ‘valence-neutral’ index of WMC to fully capture the nature of more emotionally-laden executive challenges in day-to-day cognition [25]. For example, a woman suffering from posttraumatic stress who is endeavouring to comprehend and encode a complex project briefing at work might at the same time have to struggle to set aside intrusive and distressing thoughts and memories of her trauma, thus drawing heavily on WMC resources [25]. Recently, unlike traditional RS tasks in which both memory and operation components involve emotionally-neutral material, a modified RS task was developed by Schweizer and Dalgleish [25]. This approach introduced emotionally-laden material into the operation component of the task, developing a trauma-related context as well as a valence-neutral context, to simultaneously measure WMC under a trauma-related context and WMC under a valence-neutral context for individuals with posttraumatic stress disorder (PTSD) [25]. The task required participants to remember neutral words while simultaneously processing two types of sentences: one describing emotionally-neutral facts about the world and the other describing dysfunctional trauma-related thoughts. Results showed that WMC in the context of trauma sentences, relative to WMC in the context of neutral sentences, was poorer in the PTSD group compared with the control group [25]. The results support the authors’ suggestion that people with a history of common mental health problems such as PTSD suffer from compromised WMC resources in emotional contexts, compared to healthy individuals in ways that are more marked than any such group differences manifest in valence-neutral contexts. Following Schweizer and Dalgleish [25], Shi et al. [26] further developed and tested the RS measure of WMC for test anxiety. In their work, they introduced test-related sentences as well as valence-neutral sentences into a RS task to assess WMC under a test-related context and WMC under a valence-neutral context [26]. Participants were required to perform simple tasks with emotionally benign material (i.e., remembering lists of letters) over short intervals while simultaneously dealing with emotionally-laden intrusive thoughts and feelings (i.e., processing sentences describing dysfunctional test-related thoughts) relative to emotionally-neutral information (i.e., sentences describing emotionally-neutral facts about the world) [26]. WMC under the context of sentences describing dysfunctional test-related thoughts (i.e., the ability to remember the letter lists in the context of test-related sentences), relative to WMC under the context of sentences describing emotionally-neutral facts about the world (the ability to remember the letter lists in the context of valence-neutral sentences), was poorer in high test-anxious participants compared with low test-anxious participants. The results suggested a particular difficulty employing WM in the context of sentences describing dysfunctional test-related thoughts in individuals with test anxiety, supporting the prediction of PET that it is worry intrusive thoughts that disrupt WMC [26].

As mentioned above, Ashcraft and Kirk [18] did not test the specifics of Eysenck and Calvo’s prediction (i.e., worrying intrusive thoughts detract from available WMC) but simply tested the general prediction that MA disrupts WM processing when the cognitive task involves arithmetic or math-related processes. The study did not allow for disentangling WMC impaired by worrying intrusive thoughts from WMC impaired by math-related problems involving WMC [18]. WMC in the RS task in Ashcraft and Kirk [18] involving arithmetic or math-related processes might be impaired not only by worrying intrusive thoughts induced by math-related problems but also demand on WMC by the math problems. Unlike the RS task in Ashcraft and Kirk [18], the modified RS task used in Shi et al. [26] did not involve arithmetic or math-related processes. If the modified RS task was adapted to examine the effects of MA on WMC by making stimuli (i.e., sentences describing dysfunctional math-related thoughts) specific to MA, WMC under the context of sentences describing dysfunctional math-related thoughts should not be impaired by math-related problems involving WMC but only by worrying intrusive thoughts induced by math-related sentences. This modification to the RS task would make it a suitable paradigm for examining the specifics of PET’s prediction for MA. A large number of researchers have related MA to test anxiety, suggesting that MA may be similar to test anxiety [4, 27, 28]. For instance, based on two meta-analysis studies, one including 51 studies on MA [4] and the other including 562 studies on test anxiety [29], Hembree suggested that MA and test anxiety affect performance in similar ways [4]. Therefore, it is reasonable to expect that WMC under the context of sentences describing dysfunctional math-related thoughts, relative to WMC under the context of sentences describing emotionally-neutral facts about the world, would be poorer in HMA persons compared with LMA persons.

## Materials and Methods

### Participants

One hundred and twenty-two undergraduates (mean age, 19.93 ± 1.55 years; 62 females) volunteered for this study, half of them with HMA and the other half with LMA. Participants were selected from a sample of 1323 undergraduates who were assessed for MA and test anxiety. MA was tested using the Revised Mathematics Anxiety Rating Scale (MARS-R) [30] in Chinese [31], and test anxiety was tested using the Short Form of the Test Anxiety Inventory (TAI) [32] in Chinese [33]. Informed written consent was obtained prior to data collection. The present study followed the Declaration of Helsinki and was approved by the Ethics Committee of Research Center for Learning Science, Southeast University (located in Nanjing, Jiangsu, China).

The HMA group consisted of 61 participants (age range = 18–23, *M* = 19.79, *SD* = 1.52) who scored at 1 standard deviation above the mean on the MARS-R (*M* = 83.49, *SD* = 5.20). The LMA group consisted of 61 participants (age range = 18–23, *M* = 20.08, *SD* = 1.58) who scored at 1 standard deviation below the mean on the MARS-R (*M* = 39.80, *SD* = 4.60). These cut-off points were selected because Merrell [34] suggested that a criterion of one standard deviation above or below the normative group mean is a reasonable standard for screening with rating scales and were found to approximate those used to select HMA and LMA participants, respectively, in a previous study [18]. All participants had low scores on the Chinese version of the Short Form of the TAI (HMA participants: *M* = 5.98, *SD* = 0.74; LMA participants: *M* = 5.90, *SD* = 0.77), indicating that none of them should be classified as having high test anxiety. Groups significantly differed in MA [*t*(120) = –49.12, *p* < 0.001] but not in test anxiety [*t*(120) = 0.60, *p* = 0.550].

### Procedure

The study was carried out on an individual basis in a sound-proofed laboratory room. First, participants saw instructions and practiced the task. Second, participants completed the modified RS task. Finally, participants were fully debriefed and thanked for their participation. And each participant was given a gift.

### Materials

#### MA

The MARS-R is a 24-item version of the Math Anxiety Rating Scale (MARS) [30]. This instrument measures anxiety by presenting 24 situations which may cause MA (e.g., “taking an examination in math course”). Participants decide on the level of anxiety associated with the item by providing a score on a 5-point Likert scale ranging from 1 (no anxiety) to 5 (high anxiety). The present study used the Chinese version of the MARS-R [31], which includes 21 items. The scores for this scale have shown strong internal consistency (Cronbach’s alpha = 0.93) and high 2-week test-retest reliability (*r* = 0.93).

#### Test anxiety

The Short Form of the TAI is a 5-item version of the TAI [32]. Participants are instructed to indicate on 4-point Likert scales (1 = almost never and 4 = almost always) how they generally feel and what they generally think about in test situations (e.g., “I am thinking about the consequences of possible failure.”). The present study used the Chinese version of the Short Form of the TAI [33]. The scores for this inventory have shown strong internal consistency (Cronbach’s alpha = 0.91) and high 7-day test-retest reliability (*r* = 0.91).

### The modified RS task

**Fig 1:**
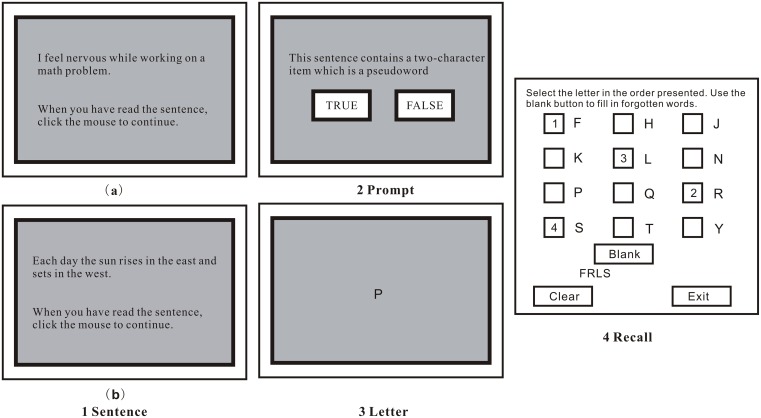
Illustration of the modified RS task. (1) A sentence (a math-related sentence [1a] or an emotionally-neutral sentence [1b]) is presented. (2) After participants read the sentence aloud, they clicked the mouse and a prompt was presented, which was judged to be “false” or “true”. (3) This was followed by a letter for 800 ms. (4) For recall, the correct letters from the current set are selected in the correct order.

In the present study, WMC under two different contexts was measured by using a modified RS task used in Shi et al. [26] (see Fig 1). Firstly, participants saw a sentence and read it aloud. Sentences were spread across math-related and valence-neutral conditions. In the former condition, in order to operationalize emotional laden intrusive thoughts and feelings, sentences were related to dysfunctional beliefs about mathematics and/or responses to it, derived from the MARS [1] (e.g., “I feel nervous while working on a math problem.”), which were used to measure WMC under the math-related context. In the latter condition, sentences were related to emotionally-neutral facts about the world (e.g., “Each day the sun rises in the east and sets in the west.”), which were used to measure WMC under the valence-neutral context. Each sentence contained 17–18 Chinese characters and the presentation order of the sentences was randomized across participants within the two conditions.

Secondly, participants clicked the mouse to advance to the next screen presenting a prompt (“This sentence contains a two-character item which is a pseudoword”) and were required to click on “True” or “False”. In order to have participants process all of the sentences as opposed to blindly responding “False” to each one, the order of two characters of a nonreversible Chinese two-character word in 25% of the sentences in each condition was reversed (e.g., “Mei Li” [a two-character word meaning beautiful] was used in a sentence as “Li Mei” [a two-character item which is a pseudoword]). The basic meanings of the sentences remained apparent even with a two-character pseudoword. Care was taken that all sentences included in the math-related and valence-neutral conditions had been coded by 20 independent undergraduates as “easy” (on a 4 point Likert scale from “easy” to “difficult”) in terms of discerning the intended meaning and as “easy” in terms of processing the sentence as containing a two-character pseudoword or not [26].

Thirdly, participants saw the letter to be recalled. After the letter disappeared, another sentence appeared, and then another letter. Given that, for HMA participants, numbers would trigger a negative emotional response and impact their performances, letters instead of numbers were used as to-be-remembered target stimuli in the modified RS task. According to Shi et al. [26], trials were of 3 sizes, comprising 3, 4 and 5 sentence-letter pairs, with 3 trials of each trial size in the modified RS task to avoid floor or ceiling effects.

Finally, a screen, listing 12 possible letters with a check box beside each one, was presented and participants were required to recall the letters in the correct serial order by clicking on the correct box. The all-or-nothing load scoring method was adopted in this study. Trials were only scored as correct if a participant recalled all of the to-be-remembered letters in the presented order. The proportions of trials correctly recalled in this manner for each trial size and for each condition were then calculated, with a higher weight assigned to correct responses on trials with a higher memory load [14]. Just like Schweizer and Dalgleish [25] and Shi et al. [26], this study used this method in order to provide some validity with regard to the math-related memory challenges. Our view was that these challenges often have an all-or-nothing quality. For instance, there is little utility in only partially remembering a mathematical formula.

## Results

### Reliability of the modified RS task

There were three presentations (i.e., trials) for each set size under the math-related and valence-neutral conditions in the modified RS task. Span scores for the first, second, and third trials under each of the two conditions were calculated. Together with the total span score, there were four estimations of span for each condition that could be used to estimate reliability [35]. The correlations between each pair of span estimations were calculated, and the average of correlations between each pair of span estimations provides a measure of task reliability. The means of the correlations for the task in the math-related and valence-neutral conditions were .84 (range .73–.93) and .82 (range .71–.92), respectively. They all indicated moderate to good task reliability [35].

### WMC

**Fig 2:**
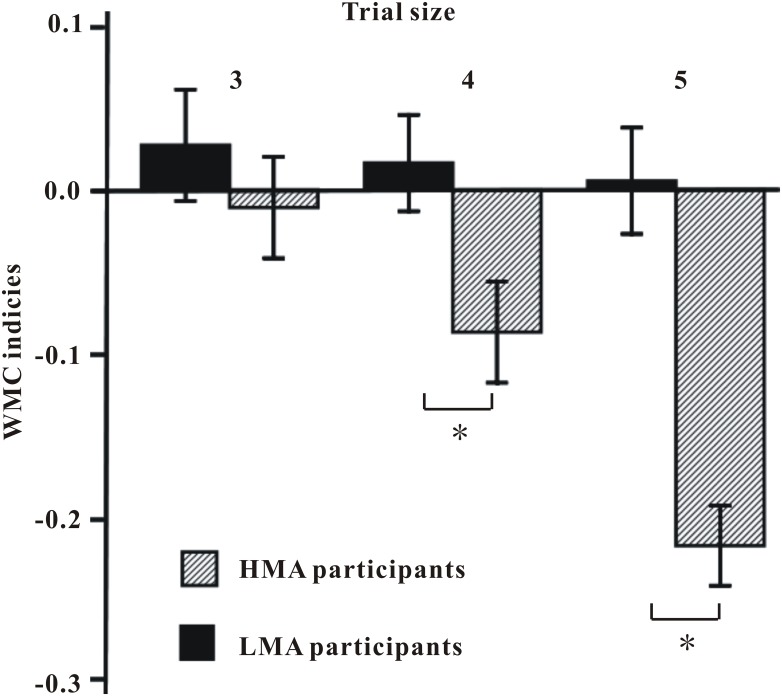
Mean (± 1SE) WMC indices (i.e., WMC scores for trials under the math-related condition minus WMC scores for trials under the valence-neutral condition) for trial sizes 3–5 for HMA participants compared with LMA participants.

WMC scores were submitted to a 2 (Group: HMA vs. LMA) × 2 (Condition: math-related vs. valence-neutral) mixed model ANOVA. The main effect of Group (*F*_(1, 120)_ = 2.00, *p* = .160, *η*_*p*_^*2*^ = .02) was not significant. The main effect of Condition (*F*_(1, 120)_ = 24.39, *p* < .0001, *η*_*p*_^*2*^ = .17) was significant, with WMC under the math-related condition (*M* = .27) being lower than WMC under the valence-neutral condition (*M* = .32). Group by Condition interaction was also significant, *F*_(1, 120)_ = 39.28, *p* < .0001, *η*_*p*_^*2*^ = .25. Further analysis showed that there were significant differences in WMC under the math-related condition, *t*(120) = 3.73, *p* < .001, Cohen's *d* = .63, but no significant differences in WMC under the valence-neutral condition, *t*(120) = −1.13, *p* = .261, Cohen's *d* = .20, between HMA and LMA participants (see Fig 2).

WMC indices (i.e., the difference in performance between math-related and valence-neutral trials) for each trial size (3, 4, 5) were entered together as dependent variables in a MANOVA with Group as independent variable. Box's test (Box's *M* = 10.53, *p* = .115) of the multivariate test for homogeneity of dispersion matrices indicated that the variances among the dependent variables are the same for the two groups; Bartlett's test of sphericity (*χ*^*2*^ = 12.24, *p* = .032) revealed that dependent variables were correlated. As this was exploratory, a statistically corrected level of alpha = .05/3 = .0167 was used for the univariate trial size analyses. For the HMA group versus the LMA group as the between-subject factor, there was a significant multivariate effect for Group, Wilk's *Lambda* = .73, *F*_(3, 118)_ = 14.47 *p* < .001, *η*_*p*_^*2*^ = .27, indicating as above that WMC indices were lower overall in the HMA group compared to the LMA group. Moreover, the univariate output showed this group difference to be significant for size 4 (marginally critical) and 5: *F*_(1, 120)_ = 5.85, *p* = .017, *η*_*p*_^*2*^ = .05; *F*_(1, 120)_ = 30.20, *p* < .001, *η*_*p*_^*2*^ = .20; not significant for size 3: *F*_(1, 120)_ = .68, *p* = .410, *η*_*p*_^*2*^ = .01 (see Fig 2).

### Reaction Time of the processing task

A 2 (Group: HMA vs. LMA) × 2 (Condition: math-related vs. valence-neutral) ANOVA was conducted with reaction time as dependent variable. Main effects of Group (*F*_(1, 120)_ = .02, *p* = .889, *η*_*p*_^*2*^ < .01) and Condition (*F*_(1, 120)_ = .09, *p* = .761, *η*_*p*_^*2*^ = .01) were not significant. Group by Condition interaction was also not significant, *F*_(1, 120)_ = 1.34, *p* = .250, *η*_*p*_^*2*^ = .01.

### Accuracy of the processing task

A 2 (Group: HMA vs. LMA) × 2 (Condition: math-related vs. valence-neutral) ANOVA was conducted with error rates as dependent variable. Main effects of Group (*F*_(1, 120)_ = 1.59, *p* = .210, *η*_*p*_^*2*^ = .01) and Condition (*F*_(1, 120)_ = .76, *p* = .386, *η*_*p*_^*2*^ = .01) were not significant. Group by Condition interaction was also not significant, *F*_(1, 120)_ = .02, *p* = .891, *η*_*p*_^*2*^ < .01.

## Discussion

The aim of this study was to examine WMC in a math-related context as well as WMC in a valence-neutral context in MA by developing a modified RS task. Consistent with the prediction, the results demonstrated that, on one hand, HMA participants performed worse on the WMC task under the context of math-related sentences (i.e., remembering the letter lists in the context of math-related sentences) than LMA participants; on the other hand, there was no group (HMA versus LMA) difference in performance on the WMC task under the context of valence-neutral sentences (remembering the letter lists in the context of valence-neutral sentences). These findings provide evidence that MA, like test anxiety [26], is characterized by impaired WMC under the context of sentences describing dysfunctional math-related thoughts. Moreover, the present study found more detailed results—WM load effects, that is, impaired WMC in MA emerged only in high WM load trials (i.e., size 4 and 5) but not in low WM load trials (size 3). This result indicated that the effects of MA on WMC were not all-or-none effects but might be accumulated effects. Additionally, no effects of MA on reaction time and accuracy were discovered in the present study.

Previous empirical studies examining the effects of MA on WMC adopted WMC tasks involving math-related problems, which resulted in confusion regarding WMC being impaired by worrying intrusive thoughts induced by math-related problems with WMC impaired by math-related problems involving WMC [18, 36, 37]. Hence, these studies were unable to disentangle the relationship between MA and WMC within the framework of PET, which claimed that the anxiety reaction involves worrying intrusive thoughts that consume the limited resources of WM, which are therefore less available for current task processing [20]. The present study of the effects of MA on WMC used the modified RS task involving math-related sentences but not involving math-related problems, which examine WMC under the context of math-related sentences rather than WMC under the context of math problems. The present findings extend previous findings by demonstrating that WMC was disrupted by math-related problems, indicate that WMC was limited by worrying intrusive thoughts induced by the context of math-related sentences, and yield support for PET. The findings also support a processing insufficiency hypothesis which was put forth by Hubbard et al. [38, 39]. This hypothesis suggested, for psychiatric persons (e.g., MA in the present study and dysphoric individuals in Hubbard et al. [38, 39]), pathology-specific cues (e.g., sentences describing dysfunctional math-related thoughts in the present study and sentences probed thoughts, feelings or behaviours related to one’s depression in Hubbard et al. [38, 39]) are maintained after cueing is removed. For these individuals, more time spent attending to pathology-specific cues in WM could result in decay of goal-relevant information and decreased performance on the WM task [38, 39]. Moreover, this processing insufficiency for goal-relevant information may extend to WM deficits when external cues are no longer present [38]. This hypothesis goes into greater detail, compared to Eysenck’s work, on the effects of pathology-specific stimuli on WMC for psychiatric groups and provides a mechanism by which capacity deficits would be observed at larger set sizes as shown in the present study.

Understanding the mechanisms of the effects of MA on mathematics performance can provide clues about how to prevent its occurrence. The current results suggest that not only reducing anxiety-induced worrying intrusive thoughts but also augmenting WMC under the context of sentences describing dysfunctional test-related thoughts in MA may be beneficial. The former mainly includes two techniques. One is expressive writing, a newly developed treatment for anxiety, which requires participants to write about their emotions for 10–15 min before a math task begins and is thought to alleviate the burden that anxiety-induced worrying intrusive thoughts place on WM by giving people a chance to re-evaluate the stressful experience [40, 41]. The other is the reappraisal or re-framing technique, which has students think positively about a test (e.g., telling students that physiological responses often associated with anxious reactions are beneficial for thinking and reasoning) and is thought to help students to reinterpret their arousal as advantageous rather than debilitating [42, 43]. Previous empirical evidence has shown that interventions reducing worrying intrusive thoughts for anxiety are valid for MA [43, 44]. However, although interventions reducing worrying intrusive thoughts for anxiety are frequently practiced [40–46], interventions augmenting emotional WMC for anxiety fall outside the purview of traditional psychological interventions. Fortunately, recent research has found that WM training under the emotional laden context could lead to transferable benefits to affective attentional processing—the emotional Stroop task and affective cognitive control—emotion regulation for healthy individuals [47, 48]. These results provide a promising platform for transferring the training techniques to clinical groups such as individuals with MA.

In a sense, the process of worrying intrusive thoughts disrupting WMC can be viewed as a processing of conflicting mental demands. The present findings provide evidence that the modified RS task, instantiating the context of sentences describing dysfunctional math-related thoughts in the task design, could not only operationalize the processing of conflicting mental demands but also better capture impaired WMC under the context of sentences describing dysfunctional math-related thoughts in MA. Future studies should use the modified RS task as a new measurement of WMC for MA to further explore the effect of MA on WMC. Moreover, in terms of the above analysis, the present findings have important educational implications. Generally, many teachers might think that a child with high MA might be less competent. However, the present findings suggest that, in terms of WM, high MA is not associated with a deficit in WM when measured using neutral information.

One limitation of the present study was that it only examined the effects of MA on verbal WM but not examined effects of MA on visual WM. In the present study, both memory and operation components in RS tasks involved verbal processing and the effects of MA on WMC reflected a conflict between the two verbal processes. The results were consistent with previous studies in which anxiety primarily impacted verbal WM [49–51], while contradicting results of Miller and Bichsel [21] in which MA, unlike other forms of anxiety, disrupts the visual WM rather than the verbal WM. Future studies should be designed to investigate the effects of MA on verbal as well as visual WM. Another limitation of this study was that it did not compare WMC under the context of math-related problems with WMC under the context of math-related sentences in MA. Given predictions of PET and the results of previous studies and the current study, WMC under the context of math-related problems should be poorer than under the context of math-related sentences because WMC under the former context was impaired not only by math-related problems involving WMC but also by worrying intrusive thoughts induced by math-related problems while WMC under the latter context was impaired only by worrying intrusive thoughts induced by math-related sentences. Distinguishing the differences between WMC in the two kinds of context in MA is necessary to understand the effects of MA on WMC and math performance. Future research should explore this issue.

In summary, the present study found impaired WMC under a math-related context in MA by adopting a modified RS task. This provided important proof-of-principal support that those suffering from MA had working memory impairments in the face of dysfunctional math-related context. The results also indicated that the modified RS task could operationalize the processing of conflicting mental demands and better capture impaired WMC under a math-related context.

## Supporting Information

S1 File The demographic data of the participants. (XLSX)

S2 File The working memory performance of participants. (XLSX)

S3 File The reaction time of participants. (XLSX)

S4 File The response accuracy of participants. (XLSX)

S5 File Span scores of trial 1, 2 and 3. (XLSX)
